# Understanding complex clinical reasoning in infectious diseases for improving clinical decision support design

**DOI:** 10.1186/s12911-015-0221-z

**Published:** 2015-11-30

**Authors:** Roosan Islam, Charlene R. Weir, Makoto Jones, Guilherme Del Fiol, Matthew H. Samore

**Affiliations:** 1 Department of Biomedical Informatics, University of Utah, 421 Wakara Way, Ste 140, Salt Lake City, UT 84108 USA; 2 IDEAS Center for Innovation, VA Salt Lake City Health System, 500 Foothill Drive, Salt Lake City, UT 84108 USA

**Keywords:** Inappropriate prescribing, Electronic health records, Decision support systems clinical, Medical informatics, Mental processes, Decision-making, Clinical complexity, Complexity in healthcare, Cognition

## Abstract

**Background:**

Clinical experts’ cognitive mechanisms for managing complexity have implications for the design of future innovative healthcare systems. The purpose of the study is to examine the constituents of decision complexity and explore the cognitive strategies clinicians use to control and adapt to their information environment.

**Methods:**

We used Cognitive Task Analysis (CTA) methods to interview 10 Infectious Disease (ID) experts at the University of Utah and Salt Lake City Veterans Administration Medical Center. Participants were asked to recall a complex, critical and vivid antibiotic-prescribing incident using the Critical Decision Method (CDM), a type of Cognitive Task Analysis (CTA). Using the four iterations of the Critical Decision Method, questions were posed to fully explore the incident, focusing in depth on the clinical components underlying the complexity. Probes were included to assess cognitive and decision strategies used by participants.

**Results:**

The following three themes emerged as the constituents of decision complexity experienced by the Infectious Diseases experts: 1) *the overall clinical picture does not match the pattern, 2) a lack of comprehension of the situation* and 3) *dealing with social and emotional pressures* such as fear and anxiety. All these factors contribute to decision complexity. These factors almost always occurred together, creating unexpected events and uncertainty in clinical reasoning. Five themes emerged in the analyses of how experts deal with the complexity. Expert clinicians frequently used 1) *watchful waiting* instead of over- prescribing antibiotics, engaged in 2) *theory of mind* to project and simulate other practitioners’ perspectives, reduced very complex cases into simple 3) *heuristics*, employed 4) *anticipatory thinking* to plan and re-plan events and consulted with peers to share knowledge, solicit opinions and 5) *seek help* on patient cases.

**Conclusion:**

The cognitive strategies to deal with decision complexity found in this study have important implications for design future decision support systems for the management of complex patients.

**Electronic supplementary material:**

The online version of this article (doi:10.1186/s12911-015-0221-z) contains supplementary material, which is available to authorized users.

## Background

Electronic Health Record (EHR) systems hold great promise for the development of Clinical Decision Support Systems (CDSS) [1]. CDSS provide intelligently filtered, patient-centered information to clinicians, potentially leading to improved performance and patient outcomes [2–5], [6, 7]. However, current CDSS tools in EHR systems may not be particularly suitable to assist with complex reasoning because they do not support both the automatic pattern matching of experts and high-level deliberative reasoning required in complex cases [8, 9]. Many different decision support tools are available for integration with the current EHR [10]. Better understanding of automatic pattern matching and analytical reasoning may guide the selection of optimum decision support tools that aid clinicians’ cognition.

Clinical reasoning is a complex process that uses cognition, metacognition and discipline-specific knowledge to gather and analyze patient information, weigh alternatives and evaluate the best possible treatment regimen [11]. In an attempt to understand complexity in clinical reasoning, Grant et al. found that physician-defined patient complexity reflects a wide range of medical, social and behavioral factors that are different from other measures of comorbidity and case-mix adjustments [12]. Rasmussen’s SRK (skill, rule, knowledge) model has also gained popularity in the human factors field [13]. According to this model, people use skill- and rule-based methods of decision-making when the task is less complex and previous experience can help. However, if the situation is unique, the decision-maker has to operate in a knowledge-based level that follows analytical processing with conceptual information. The analytical process relies heavily on mental simulation for assessing the options, hypotheses, actions and alternative plans under consideration [14]. The use of cognitive simulation helps in the generation of ideas about additional information that needs to be obtained and explains why people look for confirming evidence. Mental simulation, especially, helps to generate expectations for other cues not previously considered and guides the observation of changes in system variables [15].

Most CDSS capabilities available in commercial EHR systems (e.g., drug-drug interaction alerts) address low-level cognitive functions, such as reminding or alerting. On the other hand, expert clinicians reason at higher levels of abstraction. Therefore, the key in CDSS design is to provide users a comprehensive view that mitigates the “fog of war” without overloading them with information [16]. Systematic reviews have found that an effective CDSS must minimize the effort required by clinicians to process complex tasks [8]. To support high-level reasoning for clinicians, we need to understand the context of complex decision tasks, the interactions among task attributes and the factors that contribute to the complexity of specific decision tasks [17]. An intelligently designed CDSS and EHR can support clinical reasoning and also reduce mistakes.

Advances in CDSS are particularly necessary in the field of infectious diseases (ID). Despite some early success in seminal CDSS interventions for ID, little progress has been made to assist decision-making in this area [18, 19]. Understanding the complex decision process by ID experts may help in the design of advanced CDSS tools to help with tasks such early infection detection and treatment monitoring [20–23]. In addition, given the public health importance of ID, improvements in the understanding of cognitive strategies in ID decision- making have larger population-based implications. By understanding experts’ cognitive mechanisms, the design of future CDSS and EHR systems can incorporate explicit, unified, accurate and comprehensive mental models that match the mental workflow experts [24, 25]. Also, understanding the cognitive mechanisms to deal with the complexity can help us to support adaptive human decision-making by providing useful decision support tools to supplement rather than replace the decision-maker [26]. Understanding the cognitive mechanisms for dealing with complexity can also contribute to the design of better decision support tools for improving mental simulation. Simulation does not necessarily improve decisions, but it often increases confidence in the decision [27]. Previous studies have not looked into the underlying cognitive mechanisms used by ID experts for designing decision support tools. In this study, we seek to address the gap by understanding the cognitive strategies of ID experts for adapting to their environment while dealing with complexity. The findings can help us to provide better decision support tools to supplement clinicians’ cognition.

The overall goal for this study is to identify the constituents of decision complexity and the cognitive strategies to inform the design of health information technology and provide high-level cognitive support to clinicians. Decision complexity affects task performance and the extent and type of decision support used by individuals in decision-making. Decision-making in complex domains is considered to be a function of decision task and the expertise of the decision-maker [28]. Our study is focused on the following research questions: (1) What factors are associated with decision-making complexity experienced by ID experts? (2) What cognitive strategies do ID experts use to deal with complexity?

## Methods

### Study design

We conducted semistructured interviews with ID experts using Cognitive Task Analysis (CTA) methodology [29]. CTA is a systematic and scientific method used for studying and describing complex reasoning and knowledge that experts use to perform complex tasks [30]. CTA is an effective technique to determine the cognitive skills, strategies and knowledge required to perform tasks [29]. It is composed of several methods for understanding cognition in natural settings. Previous studies were effective in not only analyzing cognitive challenges but also eliciting the organizational challenges and environmental ambiguities of complex, time-pressured, uncertain and high-risk situations using the technique [31–34]. In this study, we have used “Combinatorics” of cognitive task analysis, which involved utilizing the critical decision method with critical incident interviews [35–37]. We have used the RATS (Relevance of study question, Appropriateness of qualitative method, Transparency of procedure and Soundness of interpretive approach) protocol for qualitative data analysis for the transcriptions of the interviews [38]. The RATS protocol provides standardized guidelines for qualitative research methods.

### Settings

The study was conducted at the Salt Lake City Veterans Administration Medical Center and the University of Utah Hospital and was approved by the Institutional Review Board (IRB). All participants provided oral informed consent that was approved by the University of Utah central IRB (IRB Name-understanding complex clinical decision tasks for better health IT design).

### Participants

Participants were 10 infectious disease experts who practice at one of the study sites. We defined “clinical expertise in infectious disease” as board certification in infectious disease, full-time work for a minimum of five years in a clinical environment and the identification by peers as an expert in the infectious disease domain. The first author contacted the participants through email and participation was voluntary. The interviews were conducted in the participants’ private offices.

### Procedure

Interviews were conducted according to the Critical Decision Method (CDM), a type of CTA [39]. The CDM procedure is described in Table 1. Each ID expert was asked to describe a recent complex case that was challenging in terms of diagnosis and/or treatment. A semistructured interview script was piloted and refined. The primary author interviewed the participants. At the end of the interviews, participants were asked to provide basic demographic information. The interviews were audio-recorded and transcribed. All identifiers were removed from the transcripts. We have included the questionnaire in the supplemental material of this paper.

**Table 1:** Critical decision method phases

Phases	Description
Incident identification and selection	The first step of the CDM process. The participant selects an appropriate incident for probing. The participant is asked to give a detailed description of the incident from the beginning to end. For example, in this study, the ID experts identified a recent case that seemed complex to solve cognitively.
Timeline verification and decision point identification	The second step is to get a clear and refined overview of the incident structure, key events and segments. For each of the key events, the participants were asked for goals at that point. For example, in this study, the timeline verification started from the very moment the ID expert got involved with the case or was referred to the case.
Progressive deepening	The third step refers to points in the timeline where the interviewer probes the participants for additional details. As a result, more details about decision points, judgments and the decision-making process are revealed. This particular phase ensures that the participants are probed for specific and detailed information regarding cognitive skills, experiences and expertise. For example, in this study the experts were asked specific questions about their gut feelings and how they knew the information that suddenly occurred to them.
“What-If” queries	In this final phase, the participants are asked hypothetical questions regarding their incidents that further help to illuminate the implicit decision-making process of the experts. For example, the interviewer asked, “If the patient had contracted a different type of pathogen, how would you have responded?”

### Data analysis

The research team conducted qualitative thematic analysis of the interview narratives [39–41]. The analysis was conducted iteratively, with three of the coauthors (RI, CRW, GDF) independently identifying relevant concepts associated with aspects of complexity, sense-making, cognitive goals and adaptive strategies.

The goal of this analysis was to identify the major categories and themes in the verbal protocol data, including the data analysis step of CDM: data preparation, data structuring, discovering meaning and representing findings [35]. Three researchers conducted the data analysis over multiple sessions. The procedure included detailed and systematic examination of individual interviews and a structured documentation process of coding. The content analysis was conduced in four phases: initial review of the data set, data coding, synthesis and grouping codes and representation by themes. The researchers coded only the statements that increased overall complexity or uncertainty for treating the patient.

From the beginning of the data analysis, the team members collaboratively reviewed initial categories, merged the similar codes and reached consensus if there was any disagreement. First, the codes were discussed in the group meetings. Three researchers then merged their codes into a refined category. In the subsequent meetings, the categories were discussed again and merged into factors that, at the end, were part of the themes.

This collaborative approach of consensus through discussion in qualitative research is rigorous and encourages richer conceptual analysis and interpretation [42]. For example, if the patient had two or more severe health conditions such as a previous accident and HIV, that part was coded as *risky patient characteristics*. Once the factors such as *unexpected outcome*, *risky patient characteristics* and *unusual case* were grouped, the multidisciplinary research team merged them under a broader theme, such as *overall clinical picture does not match pattern*.

The use of coding discussions to develop coding structures improves the content of the qualitative data analysis [43, 44]. Also, the multidisciplinary nature of the data analysis team contributes to an understanding of the data from a different perspective and thus adds reflexivity to the analysis [42]. This is an inclusive and inductive process that helped us identify the major themes present in the data set. The initial set of codes emerged after the initial data analysis.

Group consensus was sought at the end of each iteration, and the resulting codes were used in the subsequent iterations. Once all transcripts were coded, similar codes were merged based on code frequency and consensus. In turn, codes were consolidated into high-level themes using data-reduction techniques such as category sorting, in which interview segments are grouped according to content similarity [45]. The final step of the data analysis involved the identification of relationships among themes. Interconnected themes emerged from this analysis. Atlast.ti®7.0, a qualitative research software, was used to conduct the data analysis.

## Results

The ID experts had an average of 18.5 years of experience. Of the 10 ID experts, 2 were female and 8 were male.

### Factors associated with decision-making complexity

The following themes were identified from the factors contributing to decision-making complexity: 1) the *overall clinical picture does not match the pattern*, 2) a *lack of comprehension of the situation* and *3*) *dealing with social and emotional pressures*. These themes included several associated factors. For example, the *overall clinical picture does not match pattern* consisted of *unexpected outcome, risky patient characteristics* and *unusual case*. All these factors refer to situations in which the clinical manifestations of the patient do not match the recognized mental pattern of the clinician. This mismatch in the pattern matching may be the reason for increased uncertainty. In naturalistic decision-making (NDM) environments, the human mind looks for patterns that may help them select optimal courses of action and predict outcomes [46]. The complexity of the situation seems to be higher when clinicians cannot recognize a pattern that matches the patient’s case from their previous experience or training. A *lack of comprehension of the situation* includes the complexity factors of *lack of and/or conflicting indicator data, lack of evidence about treatment effectiveness, lack of diagnosis* and *gaps in physician’s knowledge*. These factors refer to the scarcity of information with clinical utility, which compromises situational awareness. The last theme of *social and emotional pressures* includes the factors *frustration/regret*, *liability and/or fear* and *multiple care provider conflict*. These factors contribute to clinicians’ anxiety with the decision-making process and the patient’s care.

Table 2 presents a detailed explanation of the constituents of complexity and example quotations from the interview. Emotions such as anxiety, fear or conflicts can also positively help clinicians evaluate better treatment options for the patient. Not all fear or anxiety is negative [47]. Fear and anxiety for the betterment of patients can motivate clinicians to improve clinical decision-making.

**Table 2:** Constituents of complexity with example quotations

Themes	Factors	Example quotations
Overall clinical picture does not match the pattern	Unexpected outcome	“So he was started on Cefotaxime. And about five days went by and he did not improve; he became more encephalopathic. He had trouble recalling not the city but the state and the country he was residing in”
	Risky patient characteristics	“So he’s on antiretroviral for his HIV. He is on two psychotropic medicines. He was in a car accident 10 years ago and had brain trauma at the time and he’s on one of the medications for improving memory”
	Unusual case	“I’ve never seen a case of Brucellae; that was my first one. I think I may have ordered a Brucellae culture once in the past and it was negative. But I thought that the case was just very strong for that. You know, TB of course is a common thing and that would be something that it could have been as well”
Lack of comprehension of the situation	Lack of and/or conflicting indicator data	“You start to get a trend, and when you get 20 min of data and you have a fever in a guy with pan resistant drugs it’s scary. When you have three days of the same guy going down for a smoke break, relaxing, chilling in his room, watching TV, you’re a lot more comfortable with the plan”
	Lack of evidence about treatment effectiveness	“We knew he had stuff everywhere at one point. He was sort of stalled in his clinical improvement. We were having some slight to moderate suspicion that there’s another pocket of infection, and what was the best imaging study to get. The problem is if you asked 10 radiologists you might have gotten 10 different answers. And what really happened is he probably got a very expensive, non-specific test that then led us to do a CAT scan”
	Lack of diagnosis	“Could he have candida endocarditis, or could he have some occult viscous rupture, like a ruptured diverticulum; something that would let all the candida in the GI tract suffuse into the peritoneal fluid where then it would grow like in a bath of mycology broth?”
	Gaps in physicians’ knowledge	“We looked at some review papers on vertebra osteomyelitis and we looked for guidelines. There’s guidelines about to be published but they’ve not yet been published so we looked for clinical trials but didn’t find much except for some vague low-grade recommendations that you should treat until epidural collection was resolved – but that was not specified what that meant, absolutely disappear versus no longer abscess versus no longer bone involvement. So that wasn’t very helpful”
Social and emotional pressures	Frustration/regret	“I also see sometimes there’s a nervousness or an anxiety about stopping so they continue but they never make clear in their own minds or in the medical record why they’re anxious, why they believe their patient deserves a longer duration of therapy than standard. And I think it’s an important exercise to at least be able to clarify in your own mind why you’re doing things differently and be able to express that and argue that”
	Liability and/or fear	“This is a guy who had in the past, recent past, been critically ill on various occasions, and when you look at his microbiology it’s terrifying frankly the number of bugs he has and the various resistance”
	Multiple care providers/conflict	“But the cardiology and the transplant team is very aware of all of these because anytime anything happens to the kidney all of their other medicines get screwed up including all the anti-rejection drugs. So they’re watching it like a hawk, you know”

#### Strategies used to deal with complexity

Five broad themes emerged from the data analysis: 1) *watchful waiting instead of prescribing antibiotics: less is more; 2) theory of mind: projection and simulation of other practitioners’ perspectives;* 3) *heuristics: using shortcut mental model to simplify problems; 4) anticipatory thinking: planning and re-planning for future events;* and 5) *seeking help: consultation with other experts for their opinions*.

### Watchful waiting instead of prescribing antibiotics: less is more

In general, expert ID physicians attempt to minimize antibiotic overuse. In this process, they use their clinical expertise and consensus among the team members as a means of seeking support for conservative treatment, such as avoiding overuse of antibiotics or watchful waiting to see if patients improve on their own. Experts engage the principle of “less is more” in clinical reasoning. For example,

*“There was nothing that I needed to do today on that patient. Now, again, if I really thought that the risk of endocarditis was high based on the fact that she had a murmur, any other signs or stigmata of endocarditis, then we would have gotten three blood cultures before starting antibiotics.”*

*“And so even if it’s inappropriate, prescribing an antibiotic is felt like you’re doing something; whereas not prescribing an antibiotic is maybe the more responsible thing to do but it’s still perceived as not doing anything. So, if there’s a complication, if someone gets an antibiotic and they have a complication like say a C diff infection then ‘Eh, it’s just a complication of the antibiotics’; whereas if you don’t treat them, trying to be responsible and not treating them but then they have a complication, let’s say their infection comes back or something else happens, then people will be like, ‘Well, why didn’t you do something about it?’ So that’s your fault whereas if they got a C diff infection that’s not really your fault – that’s just the way it goes.”*

### Theory of mind: projection and simulation of other practitioners’ perspectives

Theory of mind refers to the cognitive ability or capacity that can attribute mental states to self and others [15]. Experts project and simulate what other practitioners might think in terms of the course of treatment for the patient in order to simplify the problem for better communication. As a result, experts mentally “simulate” possible scenarios of how other clinicians might view past decisions. For example,

*“So, you know, I think nowadays, other clinicians might say, even if they don’t have HIV risk factors, you should test them. So, everybody with mono should probably have an HIV test. So maybe, we won’t just do unnecessary tests here.”*

*“You know, medicine folks would ask you, ‘Well, can we switch to oral now?’ I’m lik,e ‘No, I don’t think so.’ All the time for endocarditis, they’ll ask, ‘Can we use oral drugs? and I’m like, ‘Show me where in the world can you treat bacteremia with oral drugs, that’s where then you can treat them.’ And then the same people, if you don’t treat enough when they’re readmitting they’ll say, ‘Oh, he was insufficiently treated.’”*

### Heuristics: using shortcut mental model to simplify the problem

Experts construct heuristics to deal with complex cases in order to spare attention resources and to deal with information overload. For example,

*“I think usually we would consider stopping therapy in a patient who’s had six months of therapy total, IV and oral for vertebra osteomyelitis in the absence of retained prosthetic material. However, this is his second about to near death with the same pathogen and a very similar infection. He is tolerating the antibiotic very well. So, we’re considering now leaving him on oral suppressive antibiotics indefinitely.”*

*“So for me it’s always more important to get the right diagnosis and then to follow the guidelines if the person applies to the guidelines. So for me, I would rather initiate a really good history and physical and then secondarily, apply the guidelines to it. And we applied the guidelines and treated him as the guidelines would recommend.”*

### Anticipatory thinking: planning and re-planning for future events

Anticipatory thinking is the mental projection or simulation of potential events that may affect future decisions and outcomes; it is the “what-if” component of deliberative thought [48]. The ID participants also use a chronological method to understand the patient history in depth to predict the trajectory of the disease state. This form of sense-making of looking forward rather than retrospectively, is part of the macrocognitive process of anticipatory thinking [48]. For example,

*“I think the risk/benefit analysis then would favor continuing him on antibiotics because the risk of the antibiotics themselves is very low once he’s tolerated them for a certain amount of time. And the potential consequence is if he relapses from off course then it is very severe. So, in this circumstance, I suspect I’ll probably leave him on antibiotics for quite some time.”*

*“The question is what can be done so that the infection does not come back. The reason why that’s a question that’s fraught with some anxiety is that in this guy there is a significant downside every time you treat him with antibiotics. Every time he gets antibiotics there were complications, and I worry that there will be more complications that we might not be prepared for.”*

### Seeking help: consultation with other experts for their opinions

Our analysis found that experts strongly rely on and seek case consultation with other experts they trust. For example,

*“We have a weekly conference for the immune-compromised ID docs. We discussed his case in that conference and just reviewed everything, sought out any other opinions, any advice as to what other people might consider for evaluation or duration of therapy and tried to come up with kind of a consensus, which I think was very valuable.”*

*“But I will admit there have been multiple times since I’ve contacted clinicians I don’t know. I usually contact them through email. If I cannot get hold of them, then I email my colleagues, former mentors, ID physicians working here. I generally describe them about the complex case and ask them what they would do.”*

## Discussion

Previous studies on complexity in medicine have focused on patient factors related to complexity [12, 17, 49–53]. Different patient complexity measures have been developed based on the amount of care provided weighted by the diversity and variability of the patient [54–56]. Unlike previous research, the present study contributes to the understanding of complexity from the decision-making perspective. Our results reflect the deep cognitive mechanisms of ID experts to deal with complexity from well-established qualitative methods [29, 32, 36, 57–59]. The cognitive mechanisms found in our study have also been described in the context of the cognitive and decision science literature, including naturalistic decision-making, clinical reasoning, heuristics and mental simulation [15, 46, 60–63]. The cognitive strategies found in this study can help inform decision support designers for choosing the appropriate tools to be integrated within EHR for dealing with the complexity factors. In the following paragraphs, we discuss how these different tools can support the cognitive strategies for the clinicians to deal with complexity.

**Fig. 1:**
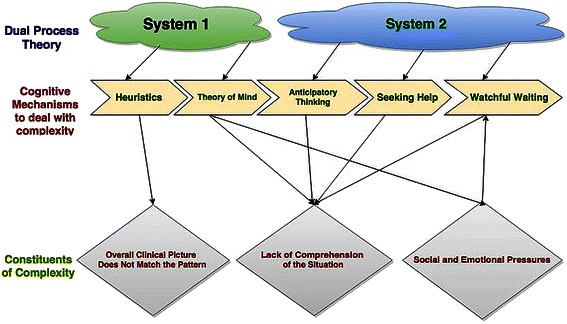
Relationship between cognitive mechanisms with constituents of complexity. The cognitive mechanisms help to deal with constituents of complexity. Only social and pressures make dealing with watchful waiting challenging. Here System 1 and System 2 refer, respectively, to automatic and analytical thinking processes

The cognitive strategies used by ID experts may help them reduce the identified complexity factors in several ways. These strategies resonate with the findings of Patterson and Woods for individuals dealing with information overload [64]. For example, *anticipatory thinking*, *theory of mind and seeking help* can support *lack of comprehension of the situation*. Risk assessment by using *anticipatory thinking* helps clinicians prioritize tasks for the best patient outcome [33, 65]. Also, *heuristics* can help when the *overall clinical picture does not match the pattern by* a short-cut mental model to fit their patients based on prior experiences [60, 63]. Moreover, w*atchful waiting* provides clinicians the time to comprehend the situation better and reduce the complexity factor of *lack of comprehension of the situation. Theory of mind* may reduce *social and emotional pressures* by group conformity and social validation. However, *social and emotional pressures* make it harder to follow a *watchful waiting*. The relationships of the cognitive strategies with the sources of decision-making complexity are shown in Fig. 1.

Dual process theory (DPT) may provide a framework to interpret the results [66]. The DPT postulates two systems of reasoning: System 1 (automatic, nonanalytic, intuitive) and System 2 (effortful, analytic, abstract and logical thinking) cognitive processes [67]^,^[68, 69]. System 2 is activated in situations associated with a high level of novelty and uncertainty, such as when complex patients are encountered. As a result, System 2 imposes significantly higher requirements for attention and cognitive effort than System 1. The cognitive mechanisms identified in this study can be interpreted as reflecting involvement of both System 1 and System 2. In fact, clinicians transition between System 1 and System 2 to efficiently adapt to the environment to deal with complexity. The mechanism of *theory of mind* requires minimal cognitive capacity and therefore is more System 1 than System 2 whereas *anticipatory thinking, seeking help* and *watchful waiting* are more aligned with the System 2 approach due to their effortful nature. Similarly, *heuristics*, which is a more automated process and thus System 1, can help when the *overall clinical picture does not match the pattern* by a short-cut mental model to fit their patients based on prior experiences [60, 63].

### Implications for decision support

**Fig. 2:**
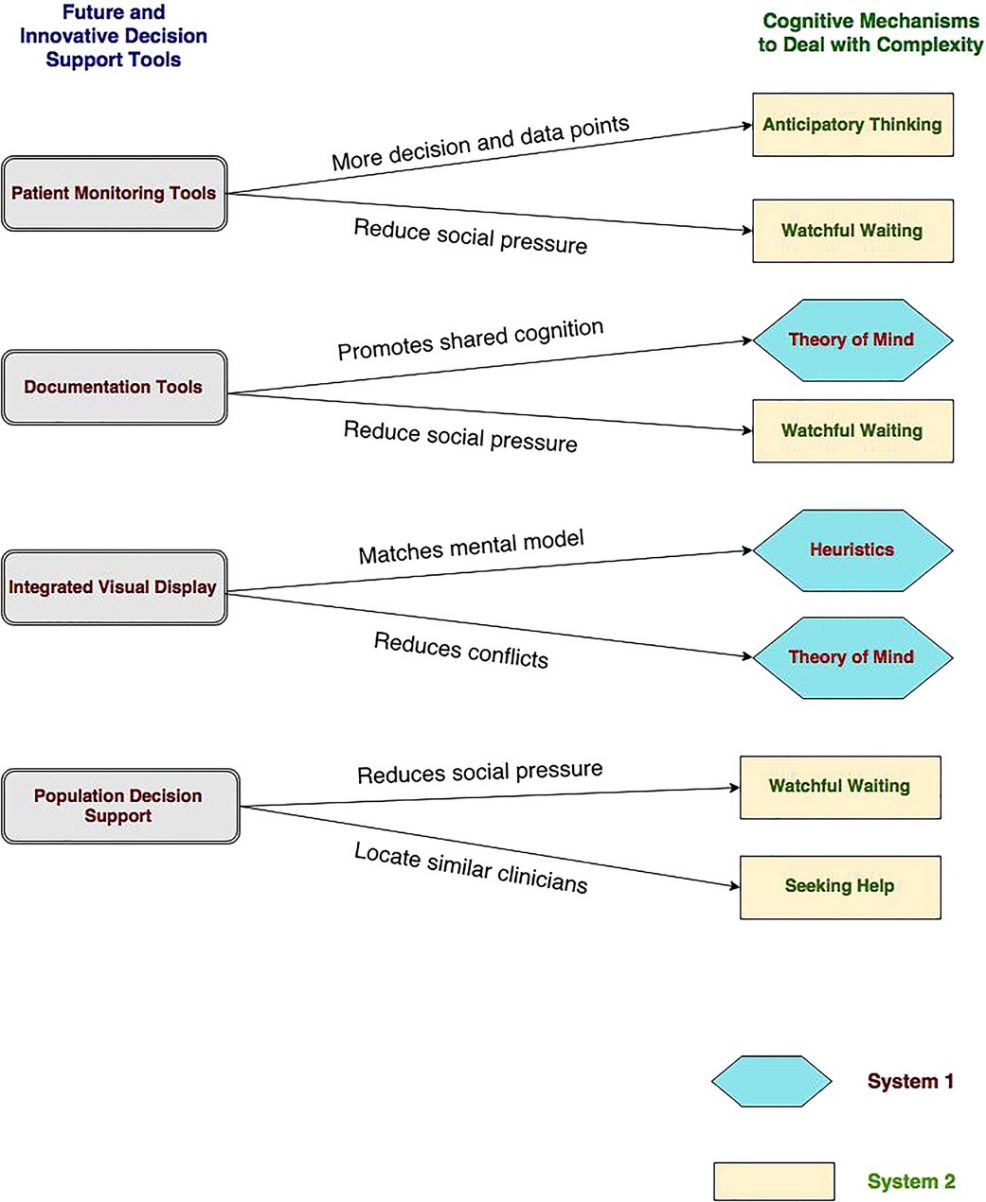
Mapping of the CDSS tools with cognitive mechanisms and dual process theory. These CDSS tools embedded in the EHR can support both System 1 and System 2 of Dual-Process Theory. Please note that just one kind of CDSS tool may not be sufficient to help with the cognitive switching

Current and future innovative informatics tools such as patient monitoring, better documentation, better visualization and population-based decision support embedded in EHR systems can facilitate clinicians’ high-level reasoning. The mapping of these tools with the cognitive strategies is illustrated in Fig. 2.

Patient monitoring tools such as therapeutic antibiotic monitors and adverse drug event monitors embedded in the EHR have the potential to support System 2 and reduce experts’ mental anxiety in *watchful waiting*. These tools also provide valuable information for *anticipatory thinking* [70–72]. For example, tele-consultation and monitoring models, such as the ECHO (Extension for Community Healthcare Outcomes) program in New Mexico, include remote patient monitoring features that may guard against or forestall potential future threats.[73] The planning and the ability to monitor the patients may help with better sense-making and preparing for the future to aid *anticipatory thinking*. In addition, these features may improve providers’ confidence in their decisions, reduce *social and emotional pressures* and as a result lead to *watchful waiting.*

Documenting decision trade-offs can reduce the fear of liability or the *social and emotional pressures* of *watchful waiting*. Also, better documentation tools that convey the rationale to support treatment decisions can make it easy for providers to understand previous decisions and goals to promote the notion of shared cognition, thereby supporting the *theory of mind* theme found in our research. Our results also suggest that supporting cognitive switching between System 1 and System 2 helps clinicians effectively manage complex clinical reasoning. For example, “Smart Forms,” a documentation-based clinical decision support system developed at Partners Healthcare, has been shown to improve decision quality and management of patients [74]. By presenting information aligned with users’ workflow, “Smart Forms” help clinicians with the automatic (System 1) thought process. At the same time, the system allows users to switch to an analytical (System 2) thought process through noninterruptive decision support recommendations for medication orders, laboratory tests, future appointments and tailored patient educational material.

Integrated visual displays can provide clinicians with information that matches the *heuristics* or the high-level mental models. In current EHR systems, information is often presented in a fragmented fashion, splitting a single patient record across multiple screens and modules in different formats. The disjointed records, redundancy of information and sheer volume of shifting data in multiple displays add a significant challenge to clinicians’ sense-making process [75–79]. Integrated displays automatically retrieve and process information from disparate modules within the EHR to provide information overview, while preserving the option of in-depth exploration on demand [80–82]. For example, a quick overview of a white board display of care coordination has been shown to improve and standardize communication in a care team in an acute care hospital [83].

Also, population-based decision support embedded in EHR systems has the potential to support System 2 with cognitive support for *seeking help* and *watchful waiting* [84]. Cognitive support to clinicians refers to maximizing clinicians’ cognitive abilities to improve clinical reasoning and decision-making with the aid of information technology [85]. Population-based decision support is a systematic application for analyzing population databases to improve the health of groups of individuals [84]. Such decision support can work as a “cognitive extension” for clinicians by providing information about treatment response for similar patients and interventions by other clinical experts. This information can help the clinician locate peer consultants who have experience with similar patients. Also, the decision not to prescribe antibiotics by other clinicians in the database can support the cognitive strategy of *watchful waiting* and reduce the associated *social and emotional* pressure.

Current information technology systems do not take the user’s emotional state into consideration. The motivational function of emotions consists of their adaptive effects on the user’s goals [47]. Therefore, for better adaptive system design, EHRs may help the clinical user reduce stress by accounting for different constituents of social and emotional pressures. For example, watchful waiting may be stressful and may increase anxiety. However, decision support tools can automatically monitor a patient’s status to help decrease the clinician’s anxiety in a watchful waiting situation.

The sociotechnical barriers that exist in our health information technology infrastructure can benefit from a better understanding of cognitive switching from System 1 to System 2 [20–23]. For example, in October 2014, a patient with Ebola virus came to a hospital in Dallas, Texas with classical symptoms of viral fever. Even though the nursing notes included travel history, it was ignored. However, an intelligently designed CDSS that encompasses local and population data could have detected the travel history as a potential threat and warned the clinicians [86]. Thus, future informatics tools that incorporate cognitive strategies into the system design may include the *heuristics* (System 1) of expert clinicians and thereby act as a cognitive extension to notify (System 2) clinicians about travel history when appropriate.

### Implications for research and practice

Overuse of antibiotics has been a concern with respect to drug resistance and public health [87, 88]. The notion that doing less in medicine sometimes can mean more has been an important discussion in the infectious disease community [89–91]. More research is needed for innovative decision support systems that can help clinicians by easing the social pressure that results from the active decision to not prescribe antibiotics.

The results of this study suggest a way to rework the paradigm of evidence-based medicine to enhance management of complex clinical tasks. Practice guidelines derived from reviews of evidence typically assume that an experienced clinician is making an assessment of the patient, which is to say that leeway for clinical judgment is allowed. However, when guidelines are incorporated into clinical decision support systems, the usual focus is to induce clinicians to accept rule-based recommendations. The role of judgment may be acknowledged, but resources are not made available to aid clinicians in reasoning through complex problems. Our hypothesis is that decision support systems should be matched to the cognitive mechanisms that clinicians use when managing complex patients. Information displays should facilitate exploration of what-if scenarios in order to improve anticipatory thinking. Better framing of the decision space would help clinicians search for appropriate heuristics and gain confidence from the experience of other clinicians.

### Limitations

The Critical Decision Method (CDM) relies on clinicians’ memory of previous cases and therefore is prone to recall bias. Also, experts possess tacit knowledge that is difficult to verbalize and articulate [29]. Thus, the CTA method is limited due to knowledge that cannot be verbalized in principle. Also, since the first author conducted the data collections, this researcher may have influenced the way the interview was conducted. To guard against this bias, we piloted and constructed the questionnaire based on the CDM instrument. In addition, our results reflect the opinions and deep cognitive processes of ID experts, which may have influenced the generalizability of the results. However, as infection is prevalent in all aspects of medicine, these results can be translated for broader impact in all areas of clinical medicine.

## Conclusion

The cognitive factors that may contribute to decision complexity include 1) *overall clinical picture does not match the pattern, 2) lack of comprehension of the situation* and 3) *social and emotional pressures.* ID experts use the following mechanisms to deal with decision complexity: 1) *watchful waiting instead of prescribing antibiotics: less is more; 2) theory of mind: projection and simulation of other practitioners’ perspectives; 3) heuristics: using shortcut mental models to simplify problems; 4) anticipatory thinking: planning and re-planning for future events; and 5) seeking help: consultation with other experts for their opinions*. Future and innovative decision support tools in the EHR may facilitate the cognitive switching from System 1 to System 2 to match experts’ high-level reasoning. CDSS and EHR designers can incorporate the cognitive mechanisms found in our study to inform the design of innovative solutions.

## Additional file

Additional file 1: **CDM ID Script.** (DOCX 94 kb)

## Abbreviations

EHR: Electronic Health Record
CDSS: Clinical Decision Support Systems
ID: Infectious Diseases
CTA: Cognitive Task Analysis
CDM: Critical Decision Method
IRB: Institutional Review Board
NDM: Naturalistic Decision Making
ECHO: Extension for Community Healthcare Outcomes

## References

[1] Jones SS, Rudin RS, Perry T, Shekelle PG (2014). Health information technology: an updated systematic review with a focus on meaningful use. Ann Intern Med.

[2] Effken JA, Brewer BB, Logue MD, Gephart SM, Verran JA (2011). Using cognitive work analysis to fit decision support tools to nurse managers’ work flow. Int J Med Inform.

[3] Horsky J, Phansalkar S, Desai A, Bell D, Middleton B (2013). Design of decision support interventions for medication prescribing. Int J Med Inform.

[4] Sintchenko V, Coiera EW (2003). Which clinical decisions benefit from automation? A task complexity approach. Int J Med Inform.

[5] Sittig DF, Wright A, Osheroff JA, Middleton B, Teich JM, Ash JS (2008). Grand challenges in clinical decision support. J Biomed Inform.

[6] Osheroff JA, Teich JM, Middleton B, Steen EB, Wright A, Detmer DE (2007). A roadmap for national action on clinical decision support. J Am Med Inform Assoc.

[7] Shaffer, J. and Coustasse, A. Computer physician order entry and clinical decision support systems: Benefits and concerns. Business and Health Administration Association Annual Conference 2012. Paper presented at the Business and Health Administration Association (BHAA) Annual Conference 2012, at the 48th Annual Midwest Business Administration Association International Conference. Chicago, Illinois. Mar 2012.

[8] Kawamoto K, Houlihan CA, Balas EA, Lobach DF (2005). Improving clinical practice using clinical decision support systems: a systematic review of trials to identify features critical to success. BMJ.

[9] Miller RA (2009). Computer-assisted diagnostic decision support: history, challenges, and possible paths forward. Adv Health Sci Educ.

[10] Welch BM, Kawamoto K (2013). Clinical decision support for genetically guided personalized medicine: a systematic review. J Am Med Inform Assoc.

[11] Simmons B (2010). Clinical reasoning: concept analysis. J Adv Nurs.

[12] Grant RW, Ashburner JM, Hong CS, Chang Y, Barry MJ, Atlas SJ (2011). Defining patient complexity from the primary care physician’s perspective: a cohort study. Ann Intern Med.

[13] Rasmussen J (1997). Risk management in a dynamic society: a modelling problem. Saf Sci.

[14] Converse S. Shared mental models in expert team decision making. Individual and group decision making: Current Issues. 1993;221

[15] Markman KD, Klein WM, Suhr JA (2012). Handbook of imagination and mental simulation.

[16] Wu HW, Davis PK, Bell DS (2012). Advancing clinical decision support using lessons from outside of healthcare: an interdisciplinary systematic review. BMC Med Inform Decis Mak.

[17] Sintchenko V, Coiera E. Decision complexity affects the extent and type of decision support use. AMIA Annu Symp Proc. 2006:724–8 PMC1839373 17238436

[18] Khabbaz RF, Moseley RR, Steiner RJ, Levitt AM, Bell BP (2014). Challenges of infectious diseases in the USA. Lancet.

[19] Tleyjeh IM, Nada H, Baddour LM (2006). VisualDx: decision-support software for the diagnosis and management of dermatologic disorders. Clin Infect Dis.

[20] Fong IW (2013). Challenges in infectious diseases.

[21] Mandl KD, Overhage JM, Wagner MM, Lober WB, Sebastiani P, Mostashari F (2004). Implementing syndromic surveillance: a practical guide informed by the early experience. J Am Med Inform Assoc.

[22] Morens DM, Folkers GK, Fauci AS (2004). The challenge of emerging and re-emerging infectious diseases. Nature.

[23] El-Kareh R, Hasan O, Schiff GD (2013). Use of health information technology to reduce diagnostic errors. BMJ Quality & Safety.

[24] Burtscher MJ, Manser T (2012). Team mental models and their potential to improve teamwork and safety: a review and implications for future research in healthcare. Saf Sci.

[25] Papp KK, Huang GC, Lauzon Clabo LM, Delva D, Fischer M, Konopasek L (2014). Milestones of critical thinking: a developmental model for medicine and nursing. Acad Med.

[26] Islam R, Weir C, Del Fiol G. Clinical complexity in medicine: a measurement model of task and patient complexity. Methods of Information in Medicine. 2015;54(5). doi:10.3414/me15-01-0031. 10.3414/ME15-01-0031 PMC4869720 26404626

[27] Roth EM, Woods DD, Pople HE (1992). Cognitive simulation as a tool for cognitive task analysis. Ergonomics.

[28] Sintchenko V, Coiera E, editors. Decision complexity affects the extent and type of decision support use. AMIA Annual Symposium Proceedings; 2006: American Medical Informatics Association. PMC1839373 17238436

[29] Hoffman RR, Militello LG (2012). Perspectives on cognitive task analysis: historical origins and modern communities of practice.

[30] Lobach DSG, Bright TJ (2012). Enabling Health Care Decisionmaking Through Clinical Decision Support and Knowledge Management. Evidence Report/Technology Assessments.

[31] Pugh CM, DaRosa DA (2013). Use of cognitive task analysis to guide the development of performance-based assessments for intraoperative decision making. Mil Med.

[32] Islam R, Weir C, Fiol GD, editors. Heuristics in managing complex clinical decision tasks in experts’ decision making. Healthcare Informatics (ICHI), 2014 IEEE International Conference on; 2014 15–17 Sept. 2014. 10.1109/ICHI.2014.32 PMC4891069 27275019

[33] Sitterding MC, Ebright P, Broome M, Patterson ES, Wuchner S (2014). Situation awareness and interruption handling during medication administration. West J Nurs Res.

[34] Thyvalikakath TP, Dziabiak MP, Johnson R, Torres-Urquidy MH, Acharya A, Yabes J (2014). Advancing cognitive engineering methods to support user interface design for electronic health records. Int J Med Inform.

[35] Crandall B, Klein GA, Hoffman RR (2006). Working minds: a practitioner’s guide to cognitive task analysis.

[36] Klein G (2000). Cognitive task analysis of teams. Cognitive Task Analysis.

[37] Craig C, Klein MI, Griswold J, Gaitonde K, McGill T, Halldorsson A (2012). Using cognitive task analysis to identify critical decisions in the laparoscopic environment. Hum Factors.

[38] Clark J (2003). How to peer review a qualitative manuscript. Peer Review in Health Sciences.

[39] Crandall B, Klein G, Hoffman R (2006). Working minds: a practitioner’s guide to cognitive task analysis.

[40] Wong BW, Blandford A. Analysing ambulance dispatcher decision making: Trialing emergent themes analysis. 2002. Ergonomics Society of Australia

[41] Hoffman RR, Crandall B, Shadbolt NR (1998). Use of the critical decision method to elicit expert knowledge: a case study in the methodology of cognitive task analysis. Hum Factors.

[42] Barry CA, Britten N, Barber N, Bradley C, Stevenson F (1999). Using reflexivity to optimize teamwork in qualitative research. Qual Health Res.

[43] Barbour RS (2001). Checklists for improving rigour in qualitative research: a case of the tail wagging the dog?. BMJ.

[44] Berends L, Johnston J (2005). Using multiple coders to enhance qualitative analysis: the case of interviews with consumers of drug treatment. Addict Res Theory.

[45] Coxon APM (1999). Sorting data: collection and analysis.

[46] Klein G (2008). Naturalistic decision making. Hum Factors.

[47] Calvo RA, D’Mello S, Gratch J, Kappas A (2014). The Oxford handbook of affective computing.

[48] Mosier KL, Fischer UM (2011). Informed by knowledge: expert performance in complex situations.

[49] Bedny GZ, Karwowski W, Bedny IS (2012). Complexity evaluation of computer-based tasks. Int J Hum Comput Interact.

[50] Franklin A, Liu Y, Li Z, Nguyen V, Johnson TR, Robinson D (2011). Opportunistic decision making and complexity in emergency care. J Biomed Inform.

[51] Jenkins DP (2009). Cognitive work analysis: coping with complexity.

[52] Kannampallil TG, Schauer GF, Cohen T, Patel VL (2011). Considering complexity in healthcare systems. J Biomed Inform.

[53] Meyer TS, Muething JZ, Lima GA, Torres BR, del Rosario TK, Gomes JO (2012). Radiological emergency response for community agencies with cognitive task analysis, risk analysis, and decision support framework. Work (Reading, Mass).

[54] Katerndahl DA, Wood R, Jaen CR (2010). A method for estimating relative complexity of ambulatory care. Ann Fam Med.

[55] Vance J, Sprivulis P (2005). Triage nurses validly and reliably estimate emergency department patient complexity. Emerg Med Australas.

[56] Shippee ND, Shah ND, May CR, Mair FS, Montori VM (2012). Cumulative complexity: a functional, patient-centered model of patient complexity can improve research and practice. J Clin Epidemiol.

[57] Crandall B, Klein G, Hoffman R (2006). Incident-based CTA: Helping practitioners “tell stories”. Working minds: a practitioners’ or ‘s guide to cognitive task analysis.

[58] Militello LG, Hutton RJ (1998). Applied cognitive task analysis (ACTA): a practitioner’s toolkit for understanding cognitive task demands. Ergonomics.

[59] Weir CR, Nebeker JJR, Hicken BL, Campo R, Drews F, LeBar B (2007). A cognitive task analysis of information management strategies in a computerized provider order entry environment. J Am Med Inform Assoc.

[60] Gigerenzer G, Hertwig R, Pachur T (2011). Heuristics: the foundations of adaptive behavior.

[61] Gigerenzer G, Kurzenhäuser S. Fast and frugal heuristics in medical decision making. Science and Medicine in Dialogue: Thinking Through Particulars and Universals. I assume this is the journal title. 2005:3–15.

[62] Gorini A, Pravettoni G (2011). An overview on cognitive aspects implicated in medical decisions. Eur J Intern Med.

[63] Kahneman D, Klein G (2009). Conditions for intuitive expertise: a failure to disagree. Am Psychol.

[64] Patterson ES, Woods DD, Tinapple D, Roth EM, editors. Using cognitive task analysis (CTA) to seed design concepts for intelligence analysts under data overload. Proceedings of the Human Factors and Ergonomics Society Annual Meeting; 2001: Sage Publications.

[65] Wegwarth O, Gaissmaier W, Gigerenzer G (2009). Smart strategies for doctors and doctors-in-training: heuristics in medicine. Med Educ.

[66] Sherman JW, Gawronski B, Trope Y. Dual-process theories of the social mind. New York Guilford Publications; 2014

[67] Dhanaraj C, Lyles MA, Steensma HK, Tihanyi L (2004). Managing tacit and explicit knowledge transfer in IJVs: the role of relational embeddedness and the impact on performance. J Int Bus Stud.

[68] Thammasitboon S, Cutrer WB (2013). Diagnostic decision-making and strategies to improve diagnosis. Curr Probl Pediatr Adolesc Health Care.

[69] Marcum JA (2012). An integrated model of clinical reasoning: dual-process theory of cognition and metacognition. J Eval Clin Pract.

[70] Timmermans DR, Sprij AJ, de Bel CE (1996). The discrepancy between daily practice and the policy of a decision-analytic model: the management of fever of unknown origin. Med Decis Making.

[71] Brown KA, Khanafer N, Daneman N, Fisman DN (2013). Meta-analysis of antibiotics and the risk of community-associated Clostridium difficile infection. Antimicrob Agents Chemother.

[72] MacKenzie A (2014). Balancing the benefits and risks of empirical antibiotics for sinusitis: a teachable moment. JAMA Intern Med.

[73] Arora S, Thornton K, Murata G, Deming P, Kalishman S, Dion D (2011). Outcomes of treatment for hepatitis C virus infection by primary care providers. N Engl J Med.

[74] Schnipper JL, Linder JA, Palchuk MB, Einbinder JS, Li Q, Postilnik A (2008). “Smart Forms” in an electronic medical record: documentation-based clinical decision support to improve disease management. J Am Med Inform Assoc.

[75] Zhang J, Norman DA (1994). Representations in distributed cognitive tasks. Cogn Sci.

[76] Zhang Y, Li Z, Wu B, Wu S (2009). A spaceflight operation complexity measure and its experimental validation. Int J Ind Ergon.

[77] Elliott AF, Davidson A, Lum F, Chiang MF, Saaddine JB, Zhang X (2012). Use of electronic health records and administrative data for public health surveillance of eye health and vision-related conditions in the United States. Am J Ophthalmol.

[78] Zhang J, Patel VL, Johnson KA, Smith JW, Malin J (2002). Designing human-centered distributed information systems. IEEE Intell Syst.

[79] Laxmisan A, Hakimzada F, Sayan OR, Green RA, Zhang J, Patel VL (2007). The multitasking clinician: decision-making and cognitive demand during and after team handoffs in emergency care. Int J Med Inform.

[80] Shneiderman B. Enabling visual discovery. Science. 2014;343(6171):614-. doi:10.1126/science.1249670.

[81] Shneiderman B (2014). Medical illuminations using evidence, visualizations and statistical thinking to improve healthcare. Science.

[82] Shneiderman B, Plaisant C, Hesse B. Improving health and healthcare with interactive visualization methods. IEEE Computer Special Issue on Challenges in Information Visualization. 2013:58–66

[83] Wong HJ, Caesar M, Bandali S, Agnew J, Abrams H (2009). Electronic inpatient whiteboards: improving multidisciplinary communication and coordination of care. Int J Med Inform.

[84] Krall MAG, Samore MH, Greenes RA (2014). Big data and population-based decision support. Clinical decison support: the road to broad adoption.

[85] Newell A, Carmichael A, Gregor P, Alm N, Waller A. Information technology for cognitive support. 2003

[86] Carroll LN, Au AP, Detwiler LT, Fu TC, Painter IS, Abernethy NF (2014). Visualization and analytics tools for infectious disease epidemiology: a systematic review. J Biomed Inform.

[87] Murray JS, Amin PM (2014). Overprescribing antibiotics in children: an enduring public health concern. J Spec Pediatr Nurs.

[88] Ascioglu S, Samore MH, Lipsitch M (2014). A new approach to the analysis of antibiotic resistance data from hospitals. Microb Drug Resist.

[89] Bell M (2014). Antibiotic misuse: a global crisis. JAMA Intern Med.

[90] Bernstein RK (2014). More can be life threatening. JAMA Intern Med.

[91] Sullivan T (2014). Antibiotic overuse and Clostridium difficile: a teachable moment. JAMA Intern Med.

