# Supplementary material for: Understanding complex clinical reasoning in infectious diseases for improving clinical decision support design
Source: BMC Med Inform Decis Mak. 2015 Nov 30;15:101. doi: 10.1186/s12911-015-0221-z (PMC4665869; doi:10.1186/s12911-015-0221-z)
Supplement: Additional file 1: — CDM ID Script. (DOCX 94 kb) [file 12911_2015_221_MOESM1_ESM.docx]

CDM ID Script:

Questions: (15 questions total)

**Incident Identification:**

1. Please narrate in details as much as you can a case that you found difficult or challenging from perspective of diagnosis or treatment uncertainty as if you are giving a report?
2. Please mention your goals at each time line and describe how you reached the goal.

**Timeline verifications:**

1. (After constructing the timeline) Is this timeline typical in your experience?
2. Please provide as much detailed information as you remember in a chronological formant.
3. Please mention goals and how you achieved the goals for each timeline?

**Deepening:**

1. What was the first decision you had to make?
   1. What information did you use when making this decision?
   2. Where did you get the information and from whom?
   3. Why did you use this information source?
2. What other courses of action were considered or were available to you at that time? How was this option chosen or others rejected? Was there a rule that you were following in choosing this option?
3. What let you know that this was the right thing to do at this point in the incident? How much time pressure were you under to make a decision? How long did it take you to actually make the decision?
4. Did you seek any guidance from other people at this point in the case? From whom? Did you trust the guidance you received? If so, why?
5. What about the decision made it particularly difficult?
6. Were you worried or surprised by any particular situation in this case?

**What-if Queries:**

1. If a novice had been in the same situation, what errors do you think they would be have committed?
2. What were some of the problems with EHR or decision-support you faced?
3. What features of decision-support tools like this can be essential for finding similar cases?
4. What kind of variables should be included in designing such interface?
